# Pediatric Isolated Sinonasal Schwannoma: A New Case Report and Literature Review

**DOI:** 10.1155/2016/2786030

**Published:** 2016-11-02

**Authors:** Xiao-Hui Ma, Hai-Chun Zhou, Can Lai, Kun Zhu, Xuan Jia

**Affiliations:** ^1^Department of Radiology, College of Medicine, The Affiliated Children's Hospital, Zhejiang University, Hangzhou, China; ^2^Department of Pathology, College of Medicine, The Affiliated Children's Hospital, Zhejiang University, Hangzhou, China

## Abstract

Schwannomas of the paranasal sinus are uncommon. Less than 4% of schwannomas involve the nasal cavity and paranasal sinuses, even less in the pediatric age group. A case of schwannoma arising in maxillary sinus in a 2.5-year-old Chinese boy is reported. The basis for discussion of this case is the exceptional rarity of sinonasal schwannoma in pediatric patients.

## 1. Introduction

Schwannomas are neoplasms that originate from Schwann cells of the neural sheath, most of which are benign and well encapsulated occurring in middle-aged adults, between the ages of 40 and 60, with an equal gender distribution [1]. Schwannomas are particularly rare in kids—merely 0.7 percent of all schwannomas took place in children during their first decade of life [2]. Computed tomography (CT) shows usually a round homogeneous density lesion, without calcification generally [3]. The cases are of interest due to the unusual arising region, uncommon pediatric patient, and the relative rarity of the radiological findings.

## 2. Case Report

A 2.5-year-old boy presented with a half-year history of nasal obstruction, rhinorrhea. There was no history of pain, fever, or trauma. Nasal stuffiness was aggravated gradually, in particular the right nasal passage, with nocturnal snoring and mouth breathing and without suffocation, headache, coma, and convulsions. Eyes secretions increased during the last 3 months, with frequent tears. The boy had a normal appetite, stool, and urine and did not have significant weight loss since the onset. A smooth surface bright red mass could be seen in the forepart of right nasal cavity with considerable purulent secretions in bilateral nasal cavity and without exophthalmos and lymphadenectasis. Bilateral external auditory canals were unobstructed, without fluid inside, and both had tympanic membrane integrity, though depression was observed in the right side. The rest of the patient's physical examinations were normal, without pigmentary changes of the skin or any other signs or symptoms of neurofibromatosis (NF); there was no family history as well.

**Figure 1:**
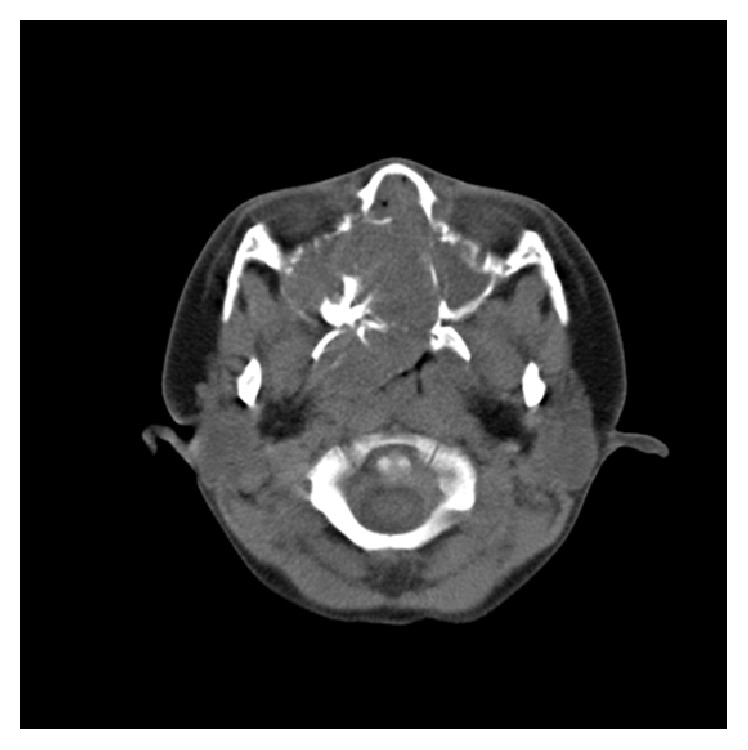
Precontrast axial CT scan shows a large round expansile mass hypoattenuating to the facial muscles and centered in the right maxillary sinus with plenty of calcifications.

**Figure 2:**
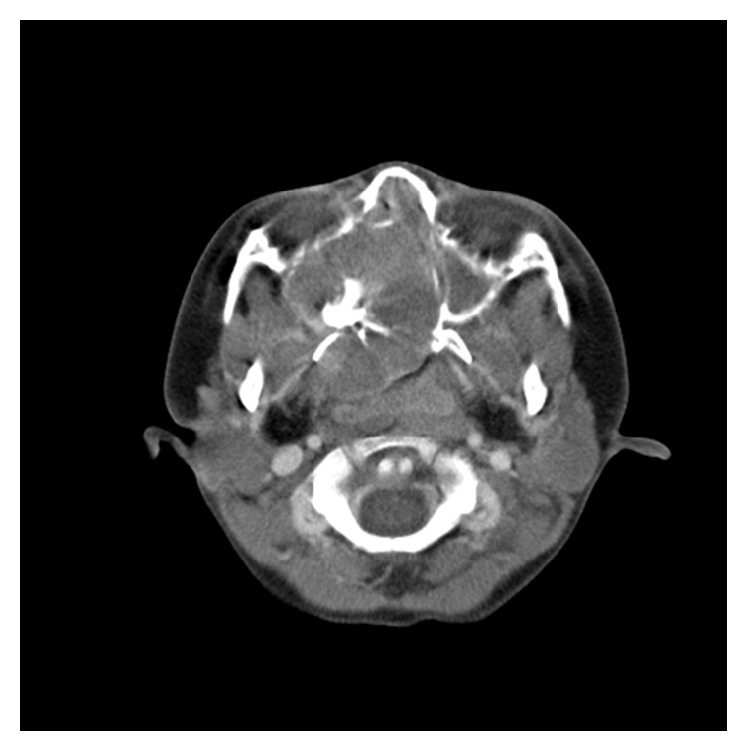
Postcontrast axial CT scan shows various portions of little enhancement within a marked heterogeneous enhancement mass and weaker intensity compared to the facial muscles.

Unenhanced CT images revealed a round (diameter = 42 mm) expansive soft-tissue mass with stellate-shaped calcification which originated from the right maxillary sinus, invaded into ethmoid sinus, sphenoid sinus region, and forepart of the right nasopharynx and oropharynx, and prolapsed into ipsilateral nasal cavity, pressuring and remodeling the adjacent bony wall of maxilla, nasal septum, and sphenoid bone with most bony margins preserved and without teeth being involved (Figure 1). The postcontrast axial CT scan showed marked heterogeneous enhancement (Figure 2)—significant enhancement (104 HU) surrounding the calcification except posterior and left part (42 HU) of the tumor, nowhere without enhancement—compared to the CT scan without contrast agent (32–50 HU).

## 3. Discussion

Schwannomas may develop virtually in all parts of the body, while the head and neck region is the most common site of origin accounting for 25% to 45% of benign schwannomas [4]. Sinonasal region, even though it is located in the head, has an extremely low incidence (about 4%) of schwannomas [5, 6] and an even lower incidence when referring to pediatric patients [2].

Schwannomas develop from any peripheral, cranial, or any autonomic nerve that has a Schwann sheath. It has been postulated that sinonasal schwannomas arise from Schwann cells of the ophthalmic and maxillary branches of the trigeminal nerve or from autonomic nerves to the septal vessels and mucosa [5, 7].

Schwannomas are usually benign neoplasms that could take place in individuals with neurofibromatosis type 2 (NF2), a kind of autosomal-dominant inherited tumor predisposition syndrome induced by NF2 gene mutations located in chromosome 22, hallmark tumor of which is the vestibular schwannoma occurring bilaterally [8]. It has been reported that patients with NF2 have developed schwannoma in the nasal cavity and paranasal sinuses [9]. All clinical examinations and tests of our patient were negative except the tumor located at paranasal sinus; additionally, he had neither family history nor any other signs or symptoms suggesting the suffering of NF; therefore, he was confirmed to have the relatively uncommon occurrence of sporadic form of schwannoma developing as a primary maxillary sinus lesion.

Sinonasal schwannoma, in which the nasal cavity and ethmoid sinus were more frequently involved than other paranasal sinuses [7], are commonly isolated, well-circumscribed masses, with nonspecific symptoms such as nasal obstruction, epistaxis, and anosmia, which expand along peripheral nerves. They appear to push rather than destroy the axons and degenerative changes; for instance, cystic alterations or hemorrhagic necrosis, are usually taking place. Since they have a tendency to grow slowly, the adjacent osseous structures remodeling can be secondary to benign pressure erosion.

CT imaging usually shows round expansile soft-tissue masses with the adjacent bony walls smoothly eroded and scalloped and with cystic or hemorrhagic degeneration often, especially in large tumors, without calcification generally [3]. These lumps are isoattenuating in comparison with the brain stem when undergoing unenhanced CT scan, while most of the tumors (88.9%) present a mild and patchy enhancement pattern after injecting contrast agent [10]. Imaging features are nonspecific and insufficient to allow a definitive diagnosis which could be confirmed only by pathological and immunohistochemical examination.

**Figure 3:**
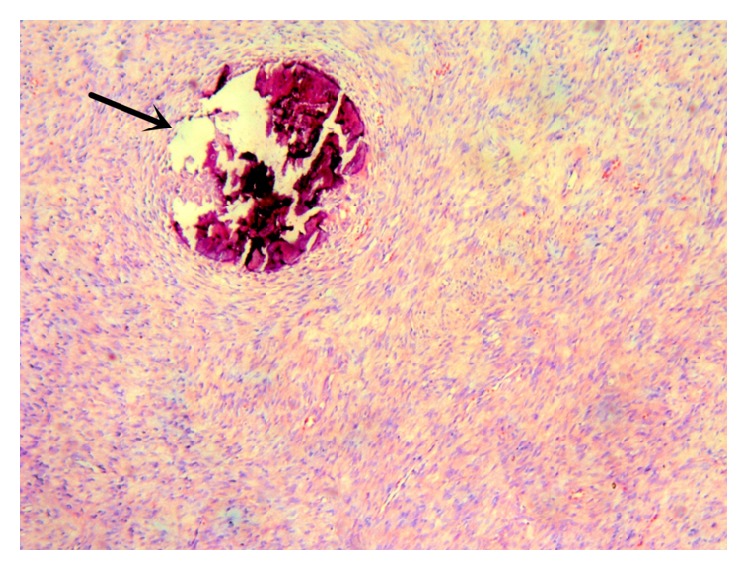
The HE stain of the lesion displayed spindle-shaped tumor cells arranged in bundles with scattered psammomatous calcifications (black arrow). H&E ×40.

Postoperative histological diagnosis confirmed that the mass was a schwannoma. In the specimen two microscopic patterns, named Antoni A and Antoni B, were seen. The characteristic of Antoni A is extended spindle-shaped cells with elongated nuclei, arranged in bundles, drifts, and whorls, and Verocay bodies, while hypocellular Antoni B areas present very loose tissue composed by polymorphism of cells and abundant myxoid. In addition, scattered psammomatous calcifications were observed in specimens (Figure 3).

The immunohistochemical study showed strong and diffuse immune response for S100 protein in tumor cells and negative response for CD34 or smooth muscle actin (SMA).

Our case is distinctive because of a number of reasons. The age of our patient is unusual as most of these cases occur between the third and fifth decades of life according to reports [11–13], and the youngest patient that has been well documented is just 10 years old [14]. Also this, to the best of our knowledge, is the only case reported with a wide range of calcification in the lesion showed by CT scan [3, 10, 15].
